# Effects of acupuncture treatment through acupoints in ears on transport stress in domestic pigs

**DOI:** 10.1093/tas/txaf030

**Published:** 2025-04-29

**Authors:** Hiroaki Kawaguchi, Moe Ijiri, Motohiko Sato, Chihiro Kanno, Hiroshi Miura, Hisaya K Ono, Yoshikazu Fujimoto, Tomohide Matsuo

**Affiliations:** Kitasato University School of Veterinary Medicine, Aomori, Japan; Joint Faculty of Veterinary Medicine, Kagoshima University, Kagoshima, Japan; Department of Surgery, Kyoto University Graduate School of Medicine, Kyoto, Japan; Kitasato University School of Veterinary Medicine, Aomori, Japan; Kitasato University School of Veterinary Medicine, Aomori, Japan; Kitasato University School of Veterinary Medicine, Aomori, Japan; Joint Faculty of Veterinary Medicine, Kagoshima University, Kagoshima, Japan; Joint Faculty of Veterinary Medicine, Kagoshima University, Kagoshima, Japan

**Keywords:** acupuncture, animal welfare, circular transdermal needles, domestic pig, swine, transport stress

## Abstract

This study aimed to investigate the effects of acupuncture treatment through ear acupoints on transport stress in domestic pigs. Experiment 1 [Safety test of new starch-circular transdermal needles (CTNs)]: Four experimental minipigs underwent the application of starch-CTNs to ear acupoints, and they were observed for 2 wk to identify any gross abnormalities. Experiment 2 (Transport stress experiment): thirty-four domestic pigs were divided into three groups: Group 1: control without treatment; Group 2: acupuncture treatment using stainless-CTNs; Group 3: acupuncture treatment using starch-CTNs. Blood samples were collected on bloodletting in the slaughterhouse, and stress and oxidative stress markers, and other biochemical factors were examined. Experiment 3 (Follow-up study): The incidence of abnormal internal organs on carcass inspection was investigated. In Experiment 1, no abnormalities were observed in any of the animals. In Experiment 2, the acupuncture treatment improved the changes in blood cortisol, noradrenaline, derivatives of reactive oxygen metabolites (d-ROMs) and biological antioxidant potential (BAP)/d-ROMs ratio. In Experiment 3, The acupuncture treatment improved rates of an abnormal digestive tract, pancreas, and heart. In conclusion, it was suggested that the acupuncture treatment through acupoints in ears using stainless- and new starch-CTNs suppresses the hypothalamic-pituitary-adrenal function, central catecholaminergic system, and affects blood oxidative stress, and consequently reduces transport stress, and can improve animal welfare for domestic pigs.

## Teaser

Acupuncture treatment through acupoints in ears using stainless- and starch-circular transdermal needles reduces transport stress and can improve animal welfare for domestic pigs.

## Graphical Abstract

**Graphical Abstract:**
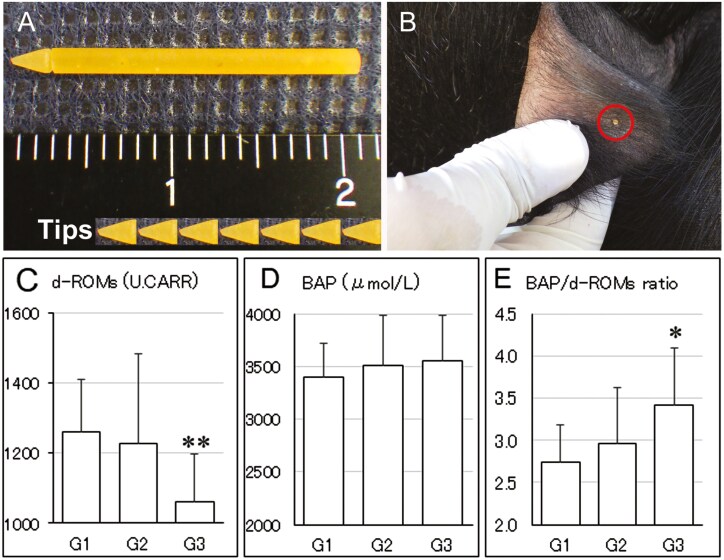

## INTRODUCTION

Stress has deleterious effects on the normal physiology of pigs, their welfare, and general productive performance (Martínez-Miró et al., 2016). It is well-known that domestic pigs are exposed to stress during transportation that increases blood cortisol levels (Dalin et al., 1993). Transport stress causes a reduction in meat quality, resulting in economic losses for farmers (Voslarova et al., 2016). Cortisol is a major glucocorticoid in pigs and increases with hypothalamic-pituitary-adrenal axis activation (Martínez-Miró et al., 2016). Transport stress depends on several factors, including crowding, temperature, duration, and fasting. To overcome the problem of transport stress, improved animal welfare for pigs is required. Therefore, we developed a minipig transport stress-reducing acupuncture treatment using a canine transport stress-reducing acupuncture treatment (Kawaguchi et al., 2016; Ijiri et al., 2023). A previous study suggested that acupuncture treatment suppresses hypothalamic-pituitary-adrenal function and, as a result, reduces transport stress without affecting the suppression of the central catecholaminergic system in minipigs (Ijiri et al. 2023). The acupuncture treatment is applied using stainless-circular transdermal needles (CTNs) to locations corresponding to acupoints in apical areas of both ears, identified as Erjian points one to three (“Jisen” in Japanese), as previously reported (Kawaguchi et al., 2016; Ijiri et al., 2023). The treatment stimulation on Jisen acupoint affects the center of the hypothalamus and vagus nerve and is considered to be affected by somatic autonomic reflexes, such as suppression and enhancement of gastric acid secretion. Generally, treatment of the apex of the ear is effective against common cold, moderate heat, poisoning, febrile disease, indigestion, and shock. Acupuncture is useful for avoiding drug residue and side effect issues in laboratory animals and livestock (Ijiri et al., 2023). However, it is difficult to ensure that stainless needles comply with hazard analysis critical control points (HACCP). Therefore, we have developed new starch-CTNs made from naturally degradable materials. This acupuncture and treatment equipment is our seed.

Domestic pigs used in this study were “Amami-Shimabuta” (indigenous middle black pig breed) and Kagoshima Berkshire on Amami-Oshima Island, located approximately 400 km south of mainland Kagoshima Prefecture in southwest Japan, raised as a regional culinary specialty (Ijiri et al., 2021). These domestic pigs are shipped from remote islands to the mainland and are transported over long periods of time by vehicle and ship, thus a method to reduce transport stress is desired.

In this study, we investigated the effects of acupuncture treatment using stainless-CTNs and new starch-CTNs on transport stress, especially regarding blood stress markers, blood oxidative stress, and carcass inspection, in “Amami-Shimabuta” pigs.

## MATERIALS AND METHODS

### Animals

All Microminipigs^TM^ were obtained from a breeder (Fuji Micra Inc., Shizuoka, Japan). All domestic pigs in a commercial pig farm on Amami-Oshima Island were used. Protocols were approved by the Ethics Committees of Animal Care and Experimentation, Kagoshima University (MD18106, MD19030). All animals were randomly selected for the experiments. The research was performed according to the Institutional Guidelines for Animal Experiments and in compliance with the Japanese Act on Welfare and Management of Animals (Act No. 105 and Notification No. 6).

### Experiment 1. Safety Test of New Starch-Circular Transdermal Needles

**Figure 1.**
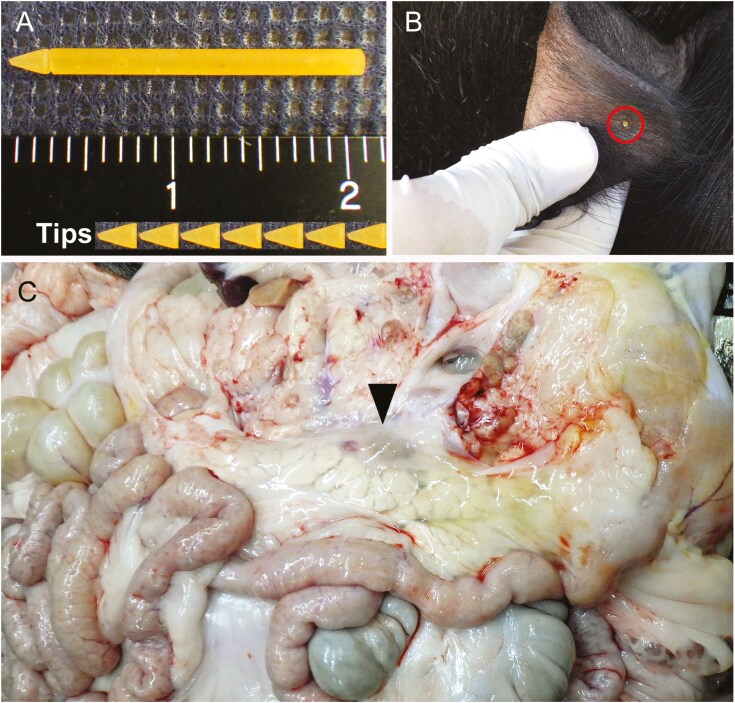
Acupuncture treatment and gross lesions of the pancreas. (A) New starch-circular transdermal needles. (B) Acupuncture treatment involving ear acupoints in a domestic pig: The tip of needles is broken and placed in the subcutaneous tissue. (C) The pancreas indicates surrounding glue-like degeneration (arrowhead) in a non-treatment domestic pig.

We have developed new starch-CTNs (original, diameter × length = 1.5 × 20 mm, Figure 1A) made from naturally degradable materials because it is difficult to ensure that stainless needles comply with HACCP. Four mature Microminipigs (2 males and 2 females, mature: over 6 mo of age, body weight: 10 to 20 kg) were used in this study. The animals underwent acupuncture corresponding to the Jisen acupoints on the apical area of both ears using starch-CTNs (original, diameter × length = 1.5 × 20 mm, Figure 1A) as previously reported (Ijiri et al., 2023). Subsequently, the treated area was observed for 2 wk to assess whether there were any gross abnormalities.

### Experiment 2. Transport Stress Experiment

Thirty-four domestic pigs (both sexes, 7 to 8 mo of age, body weight: approximately 110 kg) were divided into three groups: Group 1: control without treatment (n = 14); Group 2: acupuncture treatment using stainless-CTNs (Pyonex 1.5 mm, SEIRIN, Shizuoka, Japan, diameter × length = 0.2 × 1.5 mm) (n = 12); Group 3: acupuncture treatment using the starch-CTNs (n = 8). In the treatment groups, the animals underwent application of the acupuncture needles to the Jisen acupoints of both ears (Figure 1B), approximately 30 to 60 min before transportation as previously reported (3). All animals were transported from the production farm to the slaughterhouse in group cages (n = 3~10/cage) using a transport vehicle and ship for approximately 13 h. Acupuncture treatment was continued until slaughter.

Blood samples in the slaughterhouse were collected with a measuring cup from bloodletting, and expelled into a plane (10 ml), heparinized (10 ml), and ethylenediaminetetraacetic acid tubes (10 ml) per an animal, respectively. In the blood examination, concentrations of cortisol, catecholamines, aspartate aminotransferase, alanine aminotransferase, alkaline phosphatase, γ-glutamyl transpeptidase, creatine kinase, lactate dehydrogenase, glucose, blood urea nitrogen, creatinine, total cholesterol, triglycerides, free-cholesterol, total protein, sodium, chlorine, potassium, calcium and inorganic phosphorus, derivatives of reactive oxygen metabolites (d-ROMs) and biological antioxidant potential (BAP) were measured as previously reported (Ijiri et al., 2023; Yamada et al., 2024), and the BAP/d-ROMs ratio was also calculated.

### Experiment 3. Follow-Up Study: Incidence of Abnormal Internal Organs on Carcass Inspection

The incidence of abnormal internal organs (digestive tract, pancreas, and heart) in the pigs of Experiment 2 including animals whose blood was not sampled and the other eighty-nine pigs (as a reference) on carcass inspection was investigated (Table 1). The abnormal digestive tract included peritonitis such as fibrin precipitation in serosa, mesenteric edema, and intestinal wall thickening. The abnormal pancreas was defined mainly surrounding glue-like degeneration. In the abnormal heart, epicarditis such as fibrin precipitation in epicardium was observed. The reference investigation was conducted at the same time as Experiment 2.

**Table 1.** Incidence of abnormal internal organs on carcass inspection in Experiment 3

	Digestive tract	Pancreas	Heart
G1 (n = 25)	60.0%	12.0%	16.0%
G2 (n = 16)	56.3%	6.3%	12.5%
G3 (n = 11)	45.5%	0.0%	9.1%
Reference (n = 89)	60.7%	14.8%	15.7%

G1: control (no treatment), G2: acupuncture treatment using stainless-circular transdermal needles, G3: acupuncture treatment using starch-circular transdermal needles.

### Statistical Analysis

All data are expressed as the mean ± standard deviation (SD). Equivalence data was evaluated by Welch’s *t-*test (non-equivalence data). Incidence rates between groups were analyzed using Fisher’s exact test followed by adjustment of *p*-value using Bonferroni correction. All statistical analyses were performed using JMP Pro 17.0.0 (SAS Institute Inc., Cary, NC). The differences were considered significant at *P* < 0.05.

## RESULTS

### Experiment 1. Safety Test of Starch-Circular Transdermal Needles

No abnormalities such as hemorrhage or inflammation were observed in any minipigs for 2 wk. The tips of needles were broken and placed in subcutaneous tissue immediately after acupuncture treatment, and were excreted or had disappeared within 1 to 2 wk.

### Experiment 2. Transport Stress Experiment

**Figure 2.**
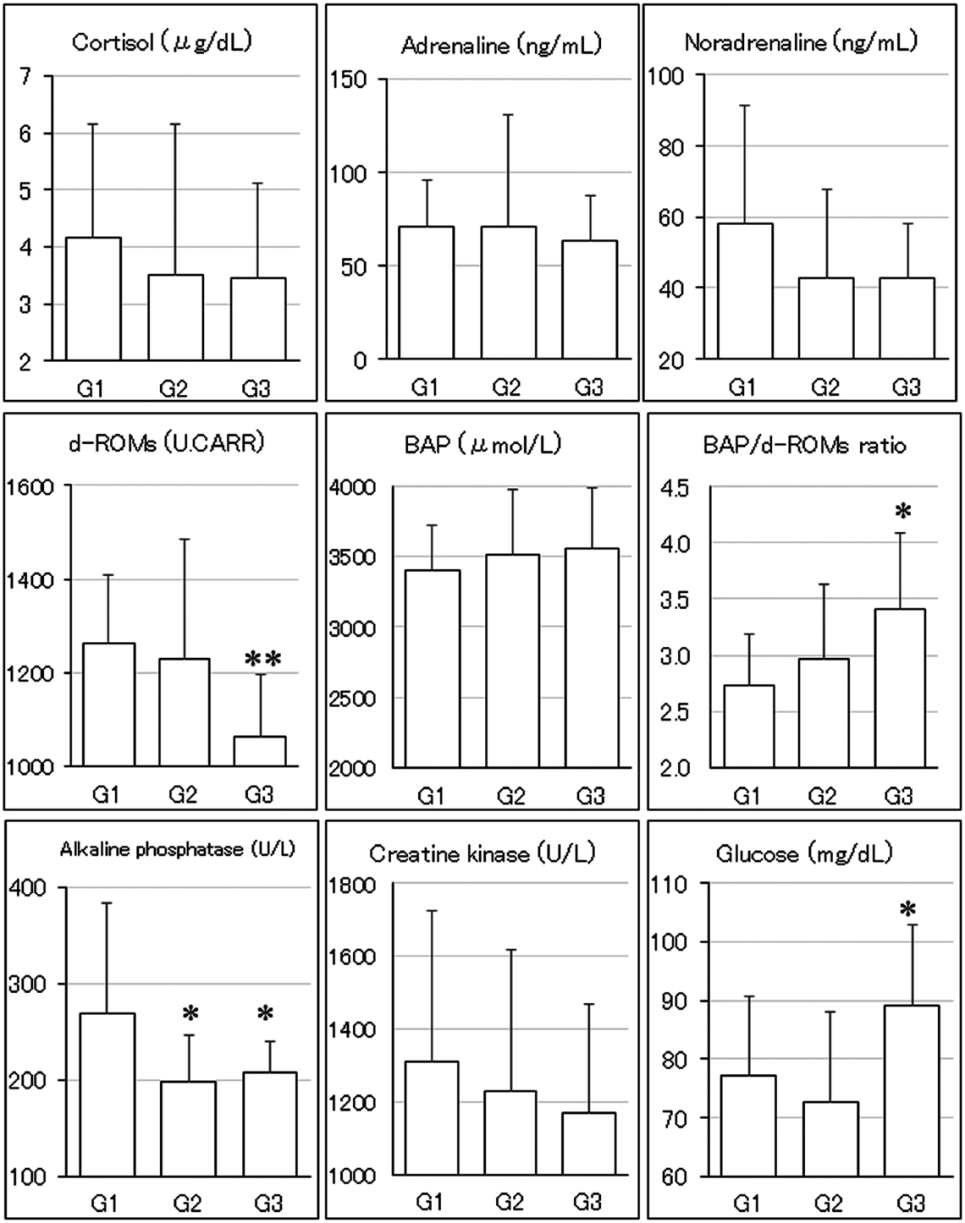
Results of blood examinations in Experiment 2. G1: control (no treatment), G2: acupuncture treatment using stainless steel-circular transdermal needles, G3: acupuncture treatment using starch-circular transdermal needles. *P < 0.05, **P < 0.01, ^#^*P* ≥ 0.05 and < 0.1 (borderline significance) vs. G1.

Data from blood examinations after transportation are shown in Figure 2. Regarding blood stress markers, cortisol concentrations in Groups 2 and 3 were lower than those in Group 1 with a reduction rate of 15.6% and 16.8% relative to Group 1, respectively. Adrenaline concentrations in Group 3 were lower than those in Group 1 with a 10.1% reduction rate relative to Group 1. Noradrenaline concentrations in Groups 2 and 3 were lower than those in Group 1 with a reduction rate of 26.1% and 26.3% relative to Group 1, respectively. Regarding blood oxidative stress, concentrations of d-ROMs in Groups 2 and 3 were lower than those in Group 1 with a reduction rate of 2.6% and 15.9% relative to Group 1, respectively. Concentrations of BAP in Groups 2 and 3 were higher than those in Group 1 with an increase rate of 3.3% and 4.6% relative to Group 1, respectively. The BAP/d-ROMs ratio in Groups 2 and 3 was higher than those in Group 1 with an increase rate of 8.4% and 24.8% relative to Group 1, respectively. Regarding other blood chemical examinations, alkaline phosphatase concentrations in Groups 2 and 3 were lower than those in Group 1 with a reduction rate of 26.1% and 22.5% relative to Group 1, respectively. Creatine kinase concentrations in Groups 2 and 3 were lower than those in Group 1 with a reduction rate of 6.2% and 10.8% relative to Group 1, respectively. Glucose concentrations in Group 3 were higher than those in Group 1 with a 15.3% increase rate relative to Group 1.

### Experiment 3. Follow-Up Study: Incidence of Abnormal Internal Organs After Slaughter

The incidence of an abnormal digestive tract, pancreas, or heart in Groups 2 and 3 was low compared with those in Group 1 and the reference (Table 1). Especially, surrounding glue-like degeneration was noted in the abnormal pancreas (Figure 1C). There were no differences in the incidence of other abnormal internal organs such as liver among the three groups. In addition, the meat quality of all “Amami-Shimabuta” pigs was normal.

## DISCUSSION

Resolving of transport stress problem in domestic animals has been required to prevent meat quality reduction and economic losses for farmers (Voslarova et al., 2016). Improvement of animal welfare is important to overcome the problem of transport stress. To the best of our knowledge, this study represents the first evaluation of acupuncture treatment using CTNs as a method of reducing transport stress in domestic pigs. The safety of acupuncture treatment using starch-CTNs was confirmed using experimental minipigs. It was not possible to collect blood from commercial pigs before shipment, and blood tests before and after transport were also not possible. The present study was a preliminary test for the practical application of the seeds verified in experimental minipigs (Ijiri et al., 2023) to domestic pigs, to proceed to large-scale research.

Data on cortisol, a typical stress marker in pigs, suggest that acupuncture treatment has a slight stress-relieving effect because blood cortisol levels after transport in animals treated with acupuncture using stainless- and starch-CTNs were slightly lower than those in non-treated animals. In both acupuncture treatments, there was a similar reduction in cortisol. The results suggest that the acupuncture treatment suppresses the hypothalamic-pituitary-adrenal function as previously reported (Ijiri et al., 2023).

We hypothesized that acupuncture treatment during transportation may relieve the extensive stress responses caused by the catecholaminergic system and contribute to improved animal well-being, while our previous study could not confirm that catecholamines are involved in transport stress (Ijiri et al., 2023). In this study, data on noradrenaline suggest that acupuncture treatment has a slight stress-relieving effect because blood noradrenaline levels after transport in animals treated with acupuncture using stainless- and starch-CTNs were lower than those in non-treated animals. The noradrenaline reduction with both acupuncture treatments was similar. The results suggest that the acupuncture treatment suppresses the central catecholaminergic system because peripheral noradrenaline levels are known to correlate with central noradrenaline levels and acupuncture modulates the activity of the locus coeruleus, which is the primary source of central noradrenergic neurons (Lee and Kim, 2017; Ziegler et al., 1977; Roy et al., 1988; Ijiri et al., 2023). Possible reasons why the central catecholaminergic system was affected differently in domestic pigs than in minipigs may be related to differences in breed and transport environment, especially the difference between group cages for domestic pigs and individual cages for minipigs but future studies are warranted to investigate this (Ijiri et al., 2023).

Although oxidative stress has detrimental effects on human health (Fukui et al., 2011; Hirata et al., 2015a, 2015b), the relationship between oxidative stress and transport stress in livestock has not been reported. It has been reported that d-ROMs are one of the blood markers to evaluate oxidative stress and chronic heat stress is a trigger for the elevation of its level in pigs without accompanied increasing general stress markers, such as cortisol (Ijiri et al., 2022). Also, BAP/d-ROMs ratio is important for antioxidant reactions to resist oxidative stress and decreases in human with obesity and metabolic syndrome (Faienza et al., 2012). However, there has been a limited number of reports describing the relationship BAP/d-ROMs ratio and swine health (Sauerwein et al., 2005; Shomo et al., 2020; Yun et al., 2020; Yamada et al., 2024). In minipigs, the acupuncture treatment reduced transport stress and, as a result, improved blood d-ROMs levels and the BAP/d-ROMs ratio since the decrease in the BAP/d-ROMs ratio owing to transport stress was caused by an increase in the d-ROMs and remaining BAP values after transport (Ijiri et al., 2023). In this study, it was considered that similar improvements in the BAP/d-ROMs ratio were achieved with acupuncture treatments using stainless- and starch-CTNs. The blood d-ROMs level and BAP/d-ROMs ratio are considered useful for evaluation of transport stress in pigs.

In this follow-up study: Incidence of abnormal internal organs on carcass inspection, A reduced incidence of an abnormal heart in animals subjected to acupuncture treatment may be related to a reduction in blood creatinine kinase levels because the meat quality of all animals was normal. Although a reduction in blood alkaline phosphatase levels was observed in animals with acupuncture treatment, there was no difference in the incidence of an abnormal liver among the three groups, and so the liver may not be affected. The surrounding glue-like degeneration in the abnormal pancreas was mainly degeneration of surrounding fat, suggesting that the degeneration is due to the nutritional status associated with fasting during transport. The acupuncture treatment may prevent degeneration caused by the nutritional status because a reduced incidence of abnormal pancreas was observed in animals with acupuncture treatment; however, future challenges remain.

## CONCLUSION

The results suggest that acupuncture treatment through Jisen acupoint in ears using stainless- and new starch-CTNs suppresses the hypothalamic-pituitary-adrenal function, central catecholaminergic system, and affects blood oxidative stress, and consequently reduces transport stress, with both types of CTNs showing similar effects in “Amami-Shimabuta” pigs. In addition, it was suggested that acupuncture treatment may also reduce internal organ abnormalities. Acupuncture treatment to lower transport-related stress can improve animal welfare for domestic pigs.
